# Personalized Stem Length Optimization in Hip Replacement: A Microscopic Perspective on Bone—Implant Interaction

**DOI:** 10.3390/bioengineering11111074

**Published:** 2024-10-27

**Authors:** Su Min Kim, Jun Won Choi, Jung Jin Kim

**Affiliations:** Department of Mechanical Engineering, Keimyung University, 1095 Dalgubeol-daero, Dalseo-gu, Daegu 42601, Republic of Korea; sm010103@gmail.com (S.M.K.); tlrp3448@gmail.com (J.W.C.)

**Keywords:** finite element analysis, optimization, proximal femur, total hip replacement

## Abstract

Total hip replacement (THR) surgery involves the removal of necrotic tissue and the replacement of the natural joint with an artificial hip joint. The demand for THR is increasing due to population aging and prolonged life expectancies. However, the uniform length and shape of artificial hip joints can cause stress shielding, leading to implant loosening and femoral fractures. These issues arise because these designs fail to account for the unique anatomical and biomechanical characteristics of individual patients. Therefore, this study proposes and validates a method to optimize stem length by considering bone microstructure and daily load. The results demonstrated that the optimal stem length varies with loading conditions and significantly reduces stress in the cortical bone while maintaining an appropriate strain energy in the cancellous bone, thereby preventing bone loss. These findings underscore the importance of patient-specific stem design for improving implant stability and clinical outcomes.

## 1. Introduction

Total hip replacement (THR) is a surgical method commonly used to treat fractures and degenerative joint diseases [1]. THR involves the removal of necrotic tissue and the replacement of the natural joint with an artificial hip joint [2]. This surgery reduces hip pain, improves joint mobility, and facilitates the activities of daily living. THR reportedly has a success rate >90%, demonstrating the high mechanical reliability of artificial hip joints [3]. THR is one of the most widely performed orthopedic surgeries worldwide [4]. Recently, the demand for THR has increased due to population aging and prolonged life expectancies [5,6].

An artificial hip joint is a medical device consisting of three main components: ball, cup, and stem [7]. The ball replaces the femoral head, whereas the cup replaces the worn-out hip socket to form a new joint. The stem is inserted into the femur and connected to the ball to replace the femur. The components of an artificial hip joint play crucial roles in replicating its natural shape and structure. The design of an artificial hip joint aims to mimic the natural bone [4]. However, issues such as femoral fractures and implant loosening still occur and must be addressed [8,9]. Specifically, the use of the shortest stems in cementless techniques prevents stress shielding but can also lead to fractures under low loads [10]. These issues arise because most artificial hip joints are manufactured with uniform lengths and shapes, which limit their ability to accommodate the unique anatomical and biomechanical characteristics of individual patients [11,12,13].

Recent studies have focused on designing artificial hip joints tailored to individual patient characteristics [14,15,16,17,18,19,20]. A custom cup and socket have been proposed to closely fit the patient’s bone shape and size with the implant geometry [16]. To improve bone ingrowth, a porous structure on the stem surface has been suggested to reduce the risk of loosening [17]. Additionally, AI-driven methods and 3D printing technologies have been employed to create personalized femoral stems and acetabular cups that accommodate the unique anatomical features of each patient [18]. Although these advancements have been significant, stem length optimization based on a patient’s unique microstructure is still lacking. Stem length is a critical factor that directly affects the surrounding cancellous bone of the femur [19,20]. This emphasizes the need to optimize stem length based on individual patient characteristics to ensure the long-term success and stability of the implant.

The stem of an artificial hip joint is a crucial component that directly interacts with the surrounding bone microstructure, thereby affecting the mechanical stability and biological integration [21,22]. The interaction between the stem and bone largely depends on the microstructure of the bone [23]. This interaction is essential to ensure proper bone ingrowth and prevent complications such as microfractures or bone resorption [24]. Individual bone microstructures in patients exhibit different anatomical patterns owing to their unique loading conditions [25]. A patient-specific approach to the stem design is required to address these individualized requirements [26]. Optimizing the stem length based on patient-specific bone microstructures and loading conditions can provide a novel approach for improving implant integration. Therefore, it is necessary to optimize the stem length tailored to the patient’s bone microstructure and loading conditions from a microscopic perspective.

This study proposes a method to optimize stem length by considering the structural behavior of the bone according to the stem implantation during daily activities. First, the skeletal image is segmented into cortical and cancellous bones for stem insertion. The stem is then implanted into a segmented image. Subsequently, a finite element model is constructed based on an image of the implanted stem. Finite element analysis and an optimization algorithm are used to iteratively evaluate the structural behavior of the femur under daily loading conditions and update the stem length. In addition, stem length optimization is performed under a multiple loading condition and compared with the results from single loading conditions.

## 2. Materials and Methods

### 2.1. Procedure for the Proposed Stem Length Optimization

**Figure 1:**
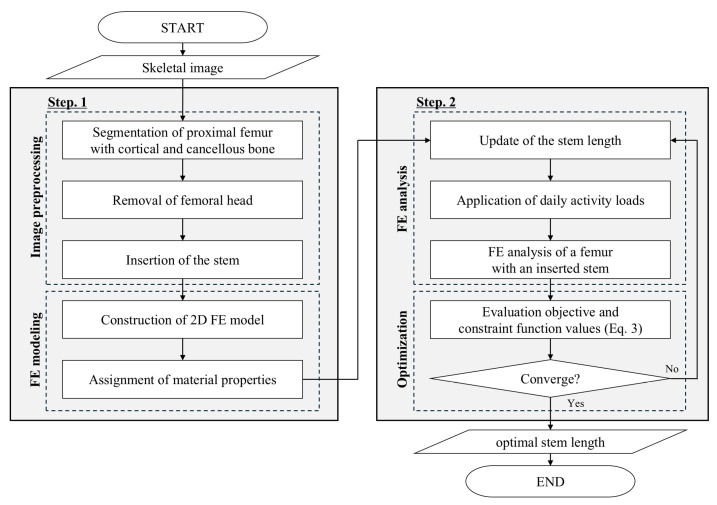
Flow chart of the proposed method.

**Figure 2:**
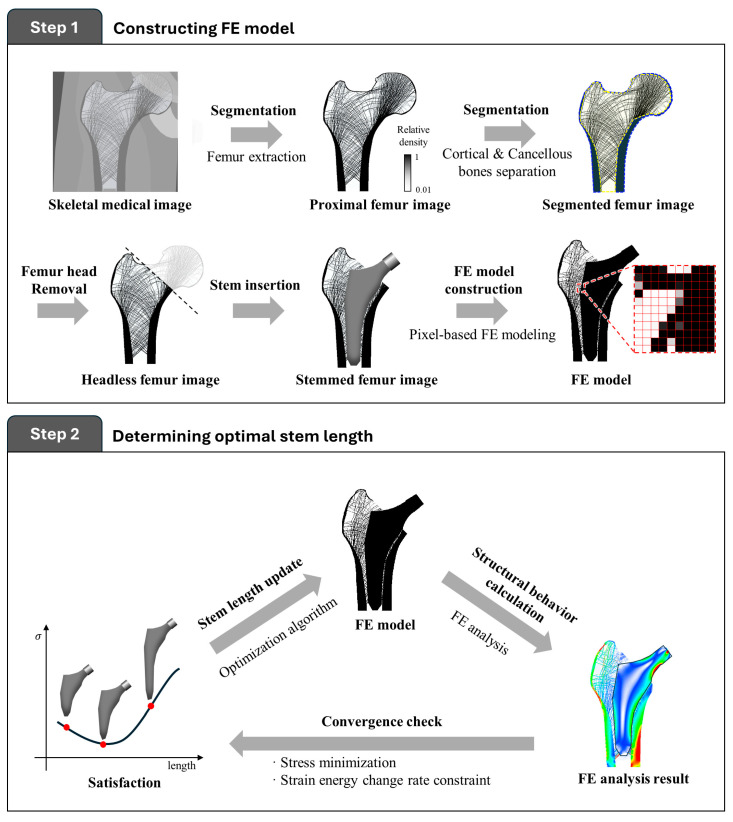
Schematic procedure of stem length optimization.

This study proposes a two-step procedure to optimize the stem length of artificial hip joints by considering the structural behavior of the bone microarchitecture during daily activities (Figure 1 and Figure 2). The first step was to construct a finite element (FE) model based on an image of the proximal femur with an artificially inserted stem. The second step was to determine the optimal stem length by iteratively analyzing the structural behavior of the bone microstructure according to stem length.

#### 2.1.1. Finite Element Modeling

This step was performed to generate an FE model from an image of a proximal femur with an artificially inserted stem. For this purpose, the preprocessing was conducted to generate a proximal femur image inserted stem. First, the proximal femur, where the artificial hip joint was to be inserted, was captured using an imaging device in clinical fields. Bone is composed of microstructures, and to obtain analysis results that closely resemble the actual structural behavior of the bone, an FE model that represents these microstructures is necessary. Thus, high-resolution images are required, with previous studies indicated that a resolution of at least 100 μm is necessary [27]. Then, only the proximal femur was extracted from the captured image containing hard tissue, soft tissue (e.g., muscle and body fat), and noise using various image-processing algorithms. Dedicated medical segmentation software, ITK (version 5.3.0) [28], and in-house codes, such as the thresholding technique [29], watershed algorithm [30], statistical shape model-based segmentation [31], and atlas-based segmentation method [32], have been frequently used in various studies. This extraction involves the segmentation of cortical and cancellous bones in the proximal femur because the bones have different mechanical properties. Specific patient characteristics (e.g., bone microstructure and shape) can be obtained from images of the femur processed through image analysis, which aids in depicting the patient’s bone more accurately when generating the femur’s FE model [33]. This ensures that the finite element analysis during the stem length optimization phase more closely reflects the structural behavior of the actual patient’s bone.

Next, the femoral head was removed, and the stem was inserted into the cancellous bone region in the extracted proximal femur image by mimicking THR. The resulting image consisted of the background, cortical bone, cancellous bone, and stem regions, with assigned values of 0, 1, 2, and 3.

Subsequently, the preprocessed image was converted into a 2D FE model using pixel-based FE modeling. This conversion accurately reflected the square shape of each pixel, represented by the four vertices in the element shape. Each pixel in the preprocessed image was converted into a square quadrilateral element with four nodes (bilinear Lagrange four-node elements), and each node had two degrees of freedom (displacements in the x- and y-directions). The element size was the same as the pixel resolution. Subsequently, the material properties of each element were assigned according to the pixel values. Elements corresponding to the pixel values of 1, 2, and 3 were assigned to the properties of the cortical bone, cancellous bone, and stem, respectively.

#### 2.1.2. Optimal Stem Length Determination

The second step was to determine the optimal stem length for an artificial hip joint subjected to the loads of daily activities. For this purpose, two processes were performed repeatedly and alternately: FE analysis and stem length updating. These two processes were terminated if the stopping criteria of the optimization algorithm were satisfied. Otherwise, the FE analysis and stem length updates continued.

**Figure 3:**
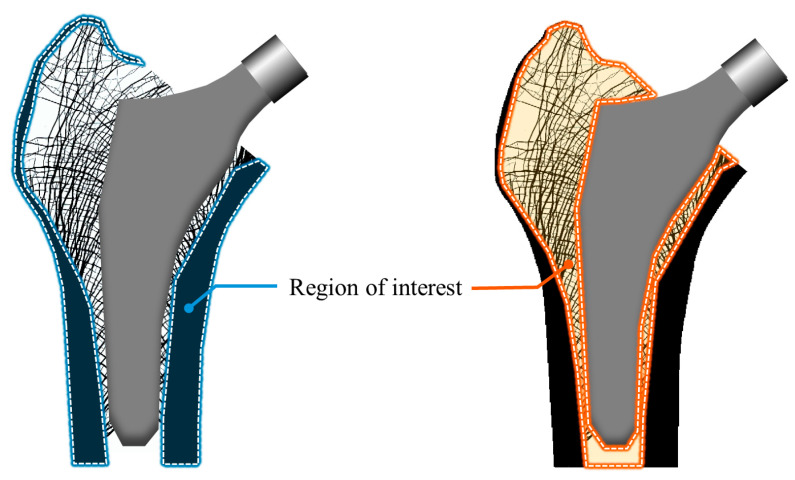
Cancellous and cortical bone, where strain energy and stress are calculated.

In the first process, an FE analysis was performed to quantify the structural behavior of the proximal femur with the implanted stem under daily activity loads. Here, daily activity loads refer to subject-specific (i.e., patient-specific) loads for the proximal femur. For example, distributed loads were applied to the surfaces of the stem head and greater trochanter, and the bottom surface of the proximal femur was fixed. Subsequently, based on the FE results, the strain energy and stress were computed for the region shown in Figure 3. Strain energy was used to evaluate the stress shielding effect in the cancellous bone [34,35,36,37,38,39,40]. When a stem is inserted into the femur, the difference in stiffness between the stem and bone leads to a reduction in strain energy within the bone [41]. According to Wolff’s law, this reduction results in bone loss [42]. Therefore, the stress shielding effect can be assessed by comparing the strain energy in the femur before and after stem insertion. The strain energy of each element corresponding to the cancellous bone was calculated as follows:

(1) $$U_{i}(k)=\frac{1}{2}{\left\{u_{i}(k)\right\}}^{T}\left[K_{i}\right]\left\{u_{i}(k)\right\}$$

where $U_{i}(k)$ denotes the strain energy of the ith element under the kth loading condition, which is calculated using the node displacement vector $u_{i}(k)$ and stiffness matrix $K_{i}$ of the element. Stress in the cortical bone, which is another representative measurement of bone structural behavior, was measured. High stresses in the bone after total hip arthroplasty may lead to postoperative complications such as pain, fractures, or dislocation, which increase the likelihood of adverse outcomes for the patient [43,44,45,46]. The stress at each node corresponding to the cortical bone is calculated as follows:

(2) $$\sigma_{i,n}(k)=\sqrt{{\left(\sigma_{i,n}^{x}(k)\right)}^{2}+{\left(\sigma_{i,n}^{y}(k)\right)}^{2}}$$

where $\sigma_{i,\;n}(k)$ denotes the stress at the nth node of ith element under the kth loading condition. $\sigma_{i,\;n}^{x}$ and $\sigma_{i,\;n}^{y}$ denote the stresses in the x and y directions.

In the second process, the stem length was updated based on the optimization formulation, as shown in Equation (3), for which satisfaction was calculated using the FE analysis results. The design variable was the stem length. The objective function was set to minimize the maximum stress in the cortical bone. Depending on the stem length, high stress may occur in the femur even under low loads, leading to postoperative complications such as fractures [10,47]. However, this study did not consider the level of stress that could cause pain. This is because it is difficult to define a fixed threshold for stress levels that cause pain, fractures, or dislocations, given the variability among patients. Note that this study was conducted based on assumptions presented in previous studies [46]. According to these assumptions, it is known that the high stress occurring in bones with an implanted stem, compared to normal bones without a stem, can lead to issues such as pain, fractures, or dislocations. Therefore, to reduce these complications, the stem length was optimized to minimize stress in the cortical bone.

The strain energy change rate in the cancellous bone was used as a constraint. If the strain energy change rate exceeds a certain threshold, bone loss due to stress shielding may occur [48]. By setting the strain energy change rate in the cancellous bone as a constraint while minimizing the stress in the cortical bone, it is possible to prevent bone loss owing to stress shielding and simultaneously minimize the maximum stress in the cortical bone. In conclusion, stem length was optimized as follows:

(3) $$\begin{array}{cc} Find & l\\ to\;minimize & f\left(l\right)=\sum_{k}c_{k}\left(\sum_{i}\sum_{n=1}^{4}\sigma_{i,n}\left(k\right)\right)\;where\;i\in S_{cor}\\ subject\;to & g\left(l\right)=\sum_{k}c_{k}\frac{1}{n}\sum_{i}\frac{U_{0,i}\left(k\right)-U_{i}\left(k\right)}{U_{0,i}\left(k\right)}\;where\;i\in S_{can}\\ & \;\;\;\;l_{min}\;\leq l\;\leq \;l_{max} \end{array}$$

where l is the length of the stem, $c_{k}$ is the weighting factor for the kth load case, $S_{cor}\;$ and $S_{can}\;$ are the sets of element numbers in the cortical and cancellous bones, respectively, n is the number of elements corresponding to the cancellous bone, $U_{0,i}(k)$ and $U_{i}(k)$ are the strain energies in the ith element of the cancellous bone before and after stem insertion in the kth load case, respectively.

### 2.2. Numerical Example for Validation

This study aimed to optimize the stem length under multiple loading conditions. To achieve this goal, a finite element model was constructed from the images of the proximal femur with the stem inserted, and the structural behavior of the proximal femur with the stem under multiple loading conditions was analyzed. Subsequently, the structural behavior was evaluated, and based on this, the stem length was updated, repeating the process until the termination criteria were satisfied.

**Figure 4:**
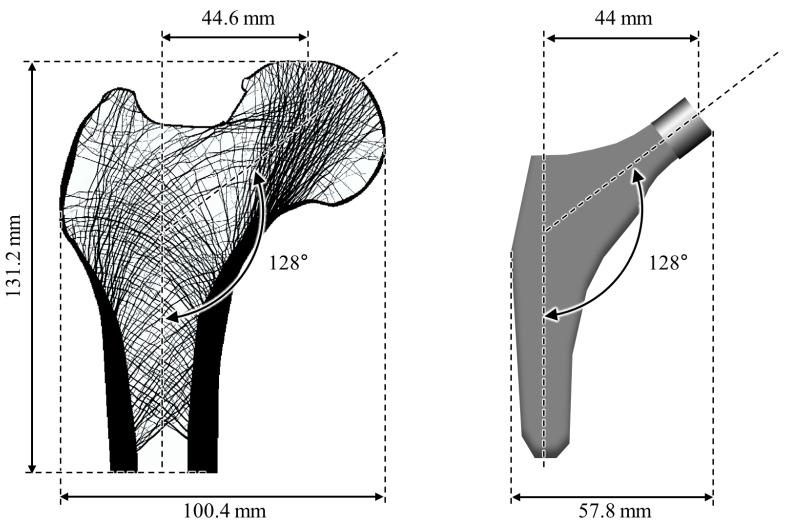
Proximal femur and stem images used in this study.

The proposed method was validated using a 50 μm high-resolution image of an artificial proximal femur obtained from previous research (Figure 4) [49]. The size of the proximal femur was 100.4 × 131.2 mm, with a femoral head offset of 44.6 mm and a neck–shaft angle of 128°. The single-wedge type was selected as the stem model intended for insertion into the artificial proximal femur. This model is widely used in clinical practice because it is structurally simple and yields excellent clinical outcomes [50]. The width of the artificial hip stem was 57.8 mm, with a head offset of 44 mm and neck–shaft angle of 128°. An image of a proximal femur with an inserted stem was created by combining the proximal femur and stem images.

An FE model was constructed from a proximal femur image with the stem image, following the steps outlined in Section 2.1, for both normal femur images and those with the implanted stem. Each pixel was converted into a four-node 2D element (PLANE 42, in ANSYS). The FE model of a normal proximal femur consisted of 2,770,997 nodes and 2,766,064 elements. The FE model of the proximal femur with an implanted stem had 2,115,241 nodes and 2,110,774 elements. Elements corresponding to the cortical bone were assigned an elastic modulus of 22.5 GPa [51]. The material properties for the stem of the superficial hip joint components were assigned a Poisson’s ratio of 0.32 and an elastic modulus of 114 GPa for titanium alloy (TiAl6V4) [48]. The Young’s modulus of the proximal femur finite element model was assigned based on the density of each element, according to Equation (4), as proposed in previous studies. A density of 0.01 was assigned to regions corresponding to the bone marrow, resulting in a very low Young’s modulus. These simulated areas were filled with soft tissues, such as the bone marrow, and the low modulus value was intended to minimize the impact of these regions on the overall structural behavior.

(4) $$\;\;E_{i}=0.3044{\left(2\rho_{i}\right)}^{1.49}E_{0}\;\;\;if\;\rho_{i}\leq 0.84\;E_{i}=0.1908{\left(2\rho_{i}\right)}^{2.39}E_{0}\;\;if\rho_{i}>0.84$$

where $\rho_{i}$ represents the bone density of the ith element and $E_{i}$ is its corresponding elastic modulus. $E_{0}$ is the reference elastic modulus of 15 GPa. The Poisson’s ratio of the cortical and cancellous bone elements was 0.3 [51]. Table 1 presents material properties used in the finite element model.

**Figure 5:**
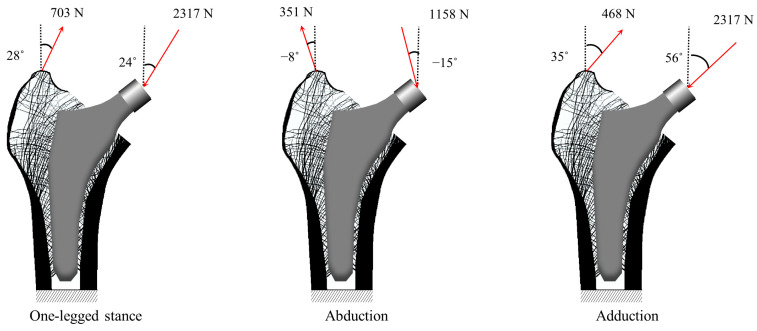
Boundary condition representing daily activities [52].

FE analysis was conducted using loading conditions from daily activities. This was achieved by applying three load cases to represent daily activities: one-legged stance, abduction, and adduction loading [52]. As shown in Figure 5, distributed loads of 2317, 1158 and 1548 N were applied to the stem head, whereas forces of 703, 351 and 468 N were applied to the intertrochanteric head. The three loading conditions used in this study represented the typical forces acting on the femur during daily activities and have been employed in several studies [49,53]. One-legged stances, abduction, and adduction naturally occur during routine activities such as walking and stair climbing. Previous studies assumed that a one-legged stance occurs at approximately 6000 cycles per day, whereas abduction and adduction occur at approximately 2000 cycles per day, leading to weightings of 0.6, 0.2, and 0.2, respectively. In addition, factors such as the patient’s body type and various recreational activities can generate different loading patterns on the hip [54], affecting both the magnitude and angle of the forces applied to the hip, which may influence stem length optimization. The lowest nodes of the proximal femur were fully constrained. In the FE analysis, the elemental behavior was assumed to be under plane stress conditions.

The golden section search method [55] was used to determine the optimal stem length using Equation (3). The golden section search method is an optimization algorithm that iteratively reduces the search interval to determine the optimal point. The design variable was the stem length. The golden section search method typically generates exact values based on the golden ratio. However, because the FE model in this study is image-based, the stem length was updated in increments of 0.05 mm, corresponding to the pixel size. The stem length was defined as the distance from the top of the stem to the lesser trochanter, 68.75 mm, and the distance to the bottom of the proximal femur model, 117.25 mm. In this study, the lower ($l_{min}$) and upper limits ($l_{max}$) of the initial interval were set to 68.75 and 117.25 mm, respectively. The convergence criteria were defined when the search interval reached 0.15 mm. The pixel size of the images used in this study was 0.05 mm. When the convergence criteria were satisfied, only one stem length remained within the search interval, which was then selected as the optimal stem length.

For the validation of the optimized stem length, this study analyzed the structural behavior by implanting the stem in the proximal femur. The behaviors were calculated by the stress and strain energy distributions under single and multiple loading conditions. All computational analyses were performed on a high-performance workstation equipped with an Intel Core™ i9-13900K processor (Intel Corporation, Santa Clara, CA, USA) and 128.0 GB of RAM, using ANSYS s2024 R1 (ANSYS, Inc., Canonsburg, PA, USA) for all FE analyses [56].

### 2.3. Compared Study

**Figure 6:**
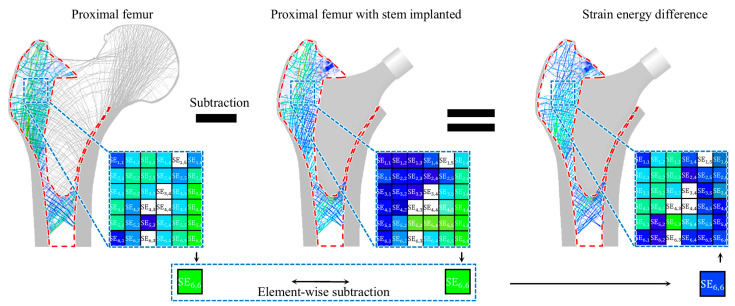
Calculation of strain energy change in the cancellous bone.

This study verified the need for stem length optimization by comparing the structural behavior of the proximal femurs implanted with stems of different lengths: the shortest, optimized, and longest stems. For this purpose, this study analyzed the structural behaviors in terms of the maximum stress and strain energy change rates under multiple loading conditions. The maximum stress was measured as the highest stress value in the cortical bone. The strain energy change rate was measured using the constraint equation from Equation (3) as the average rate of change in the strain energy for each element of the cancellous bone caused by stem insertion (Figure 6).

This study also verified the need to consider multiple loading conditions for stem length optimization. It conducted this verification by comparing the structural behaviors of proximal femurs implanted with optimized stems under different loading conditions. The stem lengths were optimized under three single loading conditions, respectively: one-legged stance, abduction, and adduction.

## 3. Results

**Figure 7:**
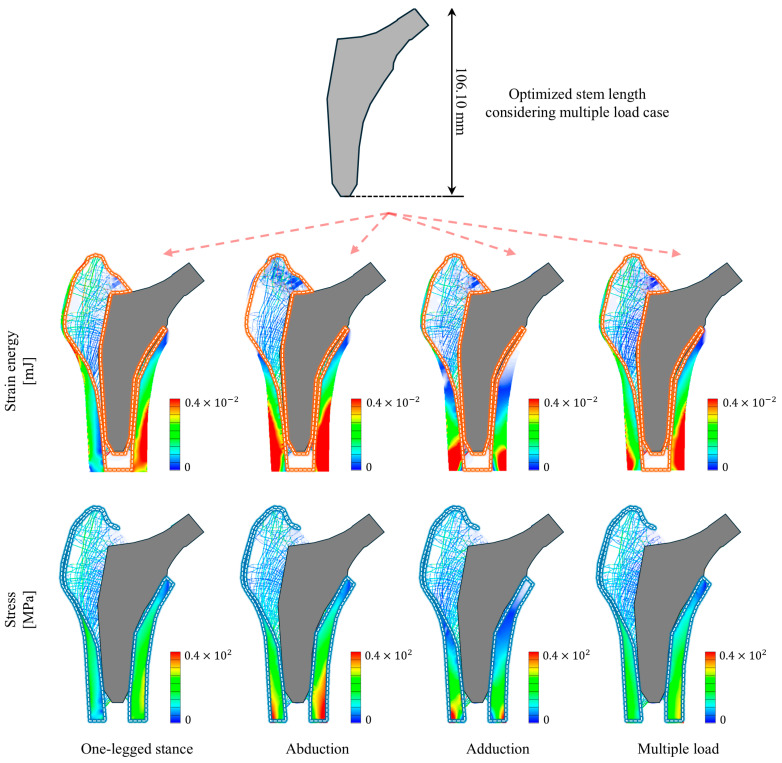
Optimized stem length under multiple loading conditions and the stress and strain energy distributions of the proximal femur with the optimized stem inserted under single and multiple loading conditions.

The optimized stem length under multiple loading conditions was 106.10 mm. Figure 7 shows the stress and strain energy distributions calculated under single- and multiple-loading conditions for the proximal femur with the optimized stem inserted. In terms of strain energy, the optimized stem exhibited a low strain energy distribution in the cancellous bone located in the femoral neck under a one-legged stance load. In contrast, under adduction and abduction loads, the optimized stem exhibited a high strain energy distribution in the cancellous bone located between the stem and lateral cortical bone. However, under multiple loading conditions, the optimized stem demonstrated a more uniform strain–energy distribution in both the cancellous bone in the femoral neck and that located between the stem and lateral cortical bone. A similar trend was observed for the stress distribution in the cortical bone. In terms of stress, the optimized stem exhibited a low stress distribution at the distal end of the lateral cortical bone under a one-legged stance load. In addition, under abduction and adduction loads, the optimized stem exhibited a high stress distribution in both the lateral and medial sides of the cortical bone. However, under multiple loading conditions, the optimized stem demonstrated a more uniform stress distribution on both the medial and lateral sides of the cortical bone.

**Figure 8:**
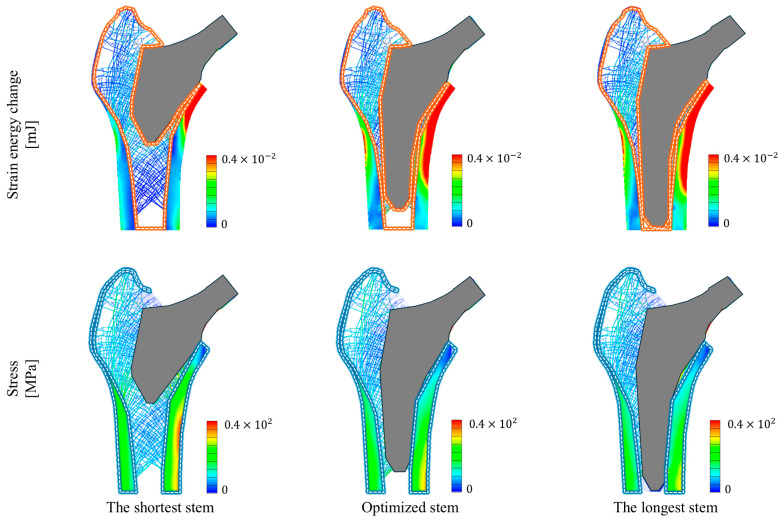
Stress and strain energy change distribution in the proximal femur under multiple loading conditions for the shortest, optimized under the multiple load condition, and longest stems.

Figure 8 presents the stress and strain energy change rates of the proximal femur under multiple loading conditions for the three stem lengths (the shortest, optimized, and longest stems). Table 2 presents the maximum stresses and strain energy change rates for the shortest, optimized, and longest stems under multiple loading conditions. Compared with the other two stems, the optimized stem exhibited a strain energy change rate that was within the values observed for the shortest and longest stems, while having the lowest maximum stress. Specifically, the strain energy change rate for the optimized stem was 27.079%, which was within the values for the shortest (9.4028%) and longest (32.127%) stems. Notably, the shortest stem caused a significant change in the strain energy in the surrounding cancellous bone, but a smaller change in the cancellous bone further from the stem, resulting in a lower strain energy change rate. In contrast, the optimized and longest stems induced a more uniform change in strain energy across the entire cancellous bone, leading to relatively higher strain energy change rates. Furthermore, the optimized stem generated a maximum stress of 101.17 MPa in the cortical bone, which was lower than those of the shortest (115.84 MPa) and longest (108.28 MPa) stems under the same loading conditions. In particular, the shortest stem concentrated stress locally in the medial cortical bone. However, both the optimized and the longest stems were found to distribute stress more evenly across a wider area of the medial cortical bone.

**Figure 9:**
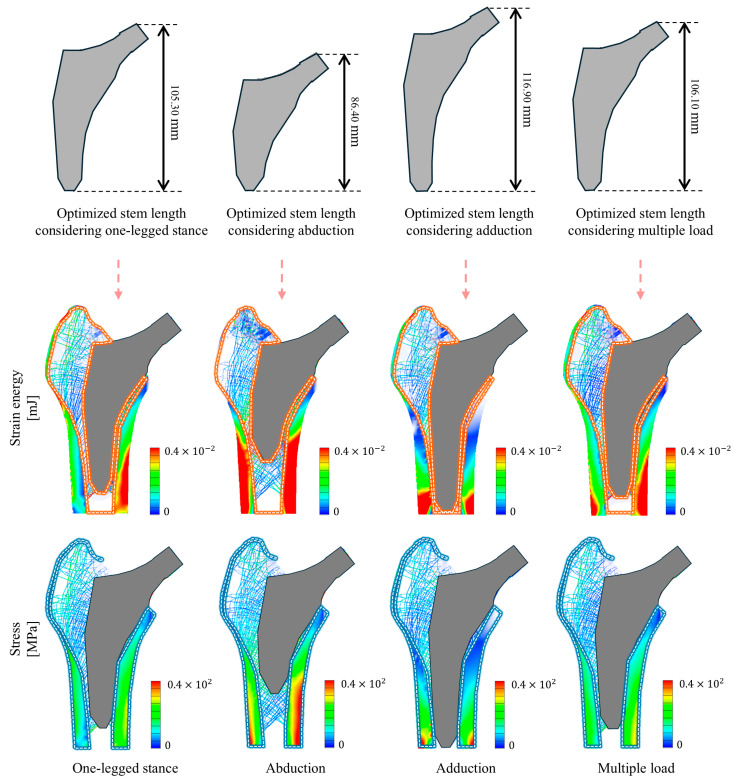
Optimized stem length and the distribution of strain energy and stress under single and multiple loading conditions.

Figure 9 shows the optimized stem lengths under single and multiple loading conditions. Additionally, Figure 9 presents the distribution of the calculated stress and strain energy change rates for the optimized stems inserted under single and multiple loading conditions. Table 3 lists the maximum stress and strain energy change rates under each loading condition when the optimized stems were inserted under single and multiple loading conditions. The optimized stem length varied depending on the loading condition in the single loading conditions. Specifically, the optimized stem length was 105.30 mm under the one-legged stance loading condition, whereas it was the shortest (86.40 mm) under the abduction loading condition. In contrast, the optimized stem length was the longest (116.90 mm) under the adduction loading condition. Notably, the optimized stem length under multiple loading conditions was 106.10 mm, which was longer than that under the one-legged stance condition, but shorter than that under the adduction condition.

The stem optimized under multiple loading conditions exhibited superior mechanical performance compared to the stem optimized under single loading conditions. For instance, the optimized stem under multiple loading conditions demonstrated a more uniform stress distribution in both the lateral and medial cortical bones than the optimized stem under single loading conditions. In addition, the maximum stress of the optimized stem under multiple loading conditions was 101.20 MPa, which was reduced by 21.2% and 44.3% compared with that under adduction and abduction loading conditions, respectively. However, it was 59.4% higher than that under the one-legged stance loading condition. In terms of the strain energy change rate, the optimized stem under multiple loading conditions had a rate of 27.079%, which was lower than that under the one-legged stance (29.034%), but higher than those under abduction and adduction (12.693% and 24.168%, respectively). Notably, the optimized stem under multiple loading conditions exhibited a more uniform strain energy distribution in the femoral trochanter than that under single loading conditions. Specifically, when compared with the optimized stems under adduction and abduction loading conditions, the optimized stem under multiple loading conditions exhibited a more uniform strain energy distribution in the cancellous bone between the stem and lateral cortical bone.

## 4. Discussion

In THR, the length of the femoral stem is a critical factor influencing implant stability and longevity. However, a standardized stem length is used in the clinical field, which may not fully account for the anatomical variations in individual patients or daily loading conditions to which the implants are subject [57]. This can result in a suboptimal load distribution between the stem and bone, increasing the risk of complications such as stem loosening, stress shielding, or even implant failure over time [58]. Therefore, this study proposed and validated a method for optimizing stem length under daily loading conditions. Moreover, this study derived the optimized stem lengths for multiple and single loads and verified the stem length optimization method by comparing the structural behavior (i.e., stress and strain energy).

This study compared the structural behavior of the shortest, optimized, and longest stems inserted into the proximal femur under multiple loading conditions. The results show that the optimized stem generated lower stress than the shortest and longest stems. This indicates that the optimized stem assists in reducing the likelihood of stress-related complications in the bone more effectively than the shortest and longest stems. In particular, under repetitive loading conditions such as those experienced during daily activities, the stem is subjected to cyclic stress that can lead to fatigue failure [59]. By reducing the stress in the cortical bone, the optimized stem lowers the likelihood of experiencing high-stress cycles that could accelerate implant wear and failure. Therefore, optimizing the stem length can minimize stress in the cortical bone, improve implant stability, and reduce the risk of damage or failure. In addition, the strain energy change rate of the optimized stem was 27.079% after insertion. This value satisfies the constraint set to prevent bone loss owing to stress shielding, confirming that the optimized stem length minimized stress while preventing bone loss.

This study confirmed that the optimal stem length for the proximal femur varied depending on single and multiple loading conditions (Table 3). The optimal stem length (105.30 mm) under the one-legged stance loading condition effectively withstood compressive forces and evenly distributed the load in the stance posture. This specific length was essential because significant stress was observed on the medial side of the proximal femur when a 24° load angle was applied to the hip implant in the one-legged stance condition. This stress pattern arises from the compressive forces transmitted to the femur when the body weight is supported by one leg. An optimized stem length of 116.90 mm under the adduction loading condition was selected to counteract the compressive forces and ensure a uniform load distribution. This specific length was crucial because significant stress concentration was observed on the medial side of the proximal femoral shaft when the leg moved toward the midline of the body at a load angle of 56°. In contrast, an optimal stem length of 86.400 mm was required under the abduction loading condition. At a load angle of −15°, this configuration generated tensile stress on the lateral side of the proximal femoral head. The selected stem length effectively counterbalanced the stress distribution, ensuring stability and reducing the risk of complications. Interestingly, when multiple loading conditions were considered, the optimal stem length differed from that derived from single loading conditions. Under multiple loading scenarios, a hip prosthesis length of 106.10 mm was required to balance the compressive and tensile forces. This difference arises because longer stems are more effective in managing compressive forces, whereas shorter stems handle tensile stress better [60]. These findings underscore the importance of customizing the hip prosthesis length by considering multiple loading conditions to improve implant performance and longevity.

Interestingly, the optimization of stem length under abduction and adduction loads had less of an effect than the one-legged stance when performed individually. Under adduction loading, the strain energy of the optimized stem increased by only 0.4% compared with that of a longer stem. The adduction loading angle was similar to the neck–shaft angle, causing load dispersion along the femoral shaft. This indicates that the strain energy changes due to the stem are minimal in the cancellous bone (Figure 8). Under abduction loading, the optimized stem reduced the stress by 1.8% compared with a shorter stem and a smaller reduction compared with other loads. When the abduction loading occurred, the force on the stem was directed toward the center of the body. This load was evenly distributed across the cortical bone through the stem. Thus, the abduction loading resulted in low-stress variations with different stem lengths. These findings emphasize the importance of designing stem lengths based on various loading conditions. They highlight the need for a stem length design that accounts for multiple loads, rather than focusing on a single load.

Interestingly, the relative rate of change in the strain energy was significantly affected by the specific loading conditions when the same stem was inserted. For example, with the shortest stem, the relative rate of change of the strain energy was the highest at 15.3% under adduction loading. In contrast, with the longest stem, the relative rate of change in the strain energy was the highest at 36.2% under a one-legged stance. These results emphasize the importance of designing the stem length based on the various loading conditions experienced by the subject. In other words, tailoring the stem length to the key multiple loading conditions that a specific patient encounters in daily life can enhance the integration and stability of the implant, which likely leads to improved long-term outcomes.

An optimized stem minimizes stress in the cortical bone, maintains strain energy in the cancellous bone, and may help prevent bone loss [61]. However, further experimental validation is necessary to confirm these effects in a clinical setting. Stress shielding after stem insertion can cause bone loss if the strain energy decreases to below a critical level. This study set the constraint for the strain energy change in the cancellous bone to <40%. However, the constraint on the strain energy change rate used in this study was an adjustable value that could be tailored for individual patients. According to previous studies, the critical value at which bone loss does not occur is generally 60% [62]. However, in this study, a more conservative value of 40% was arbitrarily set based on the findings. Further research that adjusts this constraint according to the patient’s bone microstructure or loading conditions can lead to better optimization outcomes.

This study addresses a problem similar to that addressed in previous studies. However, it makes unique contributions that enhance its originality and significance. A comparison of the stress and strain energy change based on the stem length highlights the necessity of a stem design that considers both factors [57]. In addition, the stem design should consider various patient-specific loading conditions. For example, patients who engage in intense physical activity or who have a higher body mass often experience higher loading conditions. These patients may require stem lengths that differ from those of patients with lower loads. This difference is crucial to reduce the risk of fracture and implant debonding. Based on these findings, this study demonstrates the benefits of customizing the stem length to specific patient-loading conditions. Such customization can optimize the implant stability and reduce the risk of fracture and debonding. By deriving the patient-specific optimal stem length using the method presented in this study, we expect that it can be sufficiently applied in clinical practice using modular stems with adjustable lengths [63]. Although there are limitations owing to the additional costs and time required to obtain the FE model of the femur and manufacture the stem, this approach reduces the likelihood of postoperative complications.

This study has several limitations. First, the stem length was optimized using a 2D proximal femur model. Although this approach provides useful initial insights, studies using 3D models are essential to better represent the complex anatomy and mechanical properties of the femur. Second, the optimization considered only the stem length. Other important design variables, such as the femoral neck–shaft angle and femoral head offset length, must also be considered as they significantly affect implant performance and bone integration [64]. Third, the strain energy change rate was analyzed by averaging, without separately analyzing the strain energy for each bone element. This method may lead to bone loss due to stress shielding in some areas, even if the overall constraints are met. Fourth, numerical examples contain potential sources of error. Specifically, the actual bone ingrowth between the stem and bone was not reflected, preventing the accurate representation of behaviors such as micromotion. Moreover, although real daily loads are cyclic, this study used static loads. These differences may reduce the accuracy of stem length optimization. However, despite these limitations, this study optimized the stem length for individual patients by considering both the bone microstructure and loading conditions. Future studies that address and improve these limitations could further enhance the accuracy of the optimization.

In future studies, additional analyses could be conducted in several directions to address the limitations identified in this study. This study presents a conceptual approach for optimizing stem length based on a 2D model. However, further research using 3D models is necessary to reflect actual clinical situations accurately. A 3D model can analyze the structural behavior in various directions, which is difficult to capture with a 2D model, allowing for a more precise evaluation of the stability and performance of hip implants. In future studies, we plan to use 3D models to simulate various loading conditions and surgical processes. In addition, we will refine the optimization process by considering additional design variables, such as stem thickness, shape, and neck–shaft angle. This expansion will significantly contribute to the design of customized implants tailored to the anatomical characteristics of individual patients.

## 5. Conclusions

This study demonstrates that the optimal stem length for an artificial hip joint varies depending on the loading conditions of the proximal femur where the stem is inserted. To prevent complications, such as fractures and dislocations, minimizing stress was set as the objective function. The rate of change in strain energy was used as a constraint to reduce bone loss from stress shielding. The optimized stem length obtained using this method minimized the stress under various loading conditions and prevented bone loss. These findings provide insights into the development of hip stems that reduce bone loss and stress after implantation. Incorporating this optimization approach into the design of patient-specific stems could significantly improve clinical outcomes by reducing the risk of postoperative complications such as implant loosening, fractures, and dislocations.

## Figures and Tables

**Table 1:** Material properties used in the finite element model.

Material	Young’s Modulus (GPa)	Poisson’s Ratio
Cortical Bone	22.50	0.30
Cancellous Bone *	15.00	0.30
Titanium Alloy	114.0	0.32

* Cancellous bone has a Young’s modulus of 15 GPa when ρ*_i_* is 1 according to Equation (4).

**Table 2:** Maximum stress and rate of strain energy change for the shortest stem, optimized stem, and longest stem under multiple loading conditions.

Index	Type of Hip Stem		
	The Shortest Stem	Optimized Stem	The Longest Stem
Optimized stem length [mm]	68.750	106.10	117.35
Strain energy change rate [%]	9.4028 (−65.3%)	27.079	32.127 (+18.6%)
Maximum stress [MPa]	115.84 (+14.5%)	101.17	108.28 (+7.03%)

**Table 3:** Optimized stem length, maximum stress in cortical bone, and strain energy change rate in cancellous bone under single and multiple loading conditions.

Index	Type of Loading Condition			
	One-Legged Stance	Abduction	Adduction	Multiple Load
Optimized stem length [mm]	105.30	86.400	116.90	106.10
Strain energy change rate [%]	29.034	12.693	24.168	27.079
Maximum stress [MPa]	63.461	128.34	181.75	101.17

## Data Availability

No new data were created or analyzed in this study. Data sharing is not applicable to this article.

## References

[1] Van Houcke J., Khanduja V., Pattyn C., Audenaert E. (2017). The History of Biomechanics in Total Hip Arthroplasty. Indian J. Orthop..

[2] Sculco P.K., Pagnano M.W. (2015). Perioperative Solutions for Rapid Recovery Joint Arthroplasty: Get Ahead and Stay Ahead. J. Arthroplast..

[3] Ghalme S.G., Mankar A., Bhalerao Y. (2016). Biomaterials in Hip Joint Replacement. Int. J. Mater. Sci. Eng..

[4] Colic K., Sedmak A. (2016). The Current Approach to Research and Design of the Artificial Hip Prosthesis: A Review. Rheumatol. Orthop. Med..

[5] Shan L., Shan B., Graham D., Saxena A. (2014). Total Hip Replacement: A Systematic Review and Meta-Analysis on Mid-Term Quality of Life. Osteoarthr. Cartil..

[6] Mealy A., Sorensen J. (2020). Effects of an Aging Population on Hospital Costs Related to Elective Hip Replacements. Public Health.

[7] De Santis R., Gloria A., Ambrosio L. (2017). Composite Materials for Hip Joint Prostheses. Biomedical Composites.

[8] Shahzamanian M.M., Banerjee R., Dahotre N.B., Srinivasa A.R., Reddy J.N. (2023). Analysis of Stress Shielding Reduction in Bone Fracture Fixation Implant Using Functionally Graded Materials. Compos. Struct..

[9] Lindahl H., Malchau H., Herberts P., Garellick G. (2005). Periprosthetic Femoral Fractures: Classification and Demographics of 1049 Periprosthetic Femoral Fractures from the Swedish National Hip Arthroplasty Register. J. Arthroplast..

[10] Klasan A., Bäumlein M., Dworschak P., Bliemel C., Neri T., Schofer M.D., Heyse T.J. (2019). Short Stems Have Lower Load at Failure than Double-Wedged Stems in a Cadaveric Cementless Fracture Model. Bone Jt. Res..

[11] Moscol I., Solórzano-Requejo W., Ojeda C., Rodríguez C. Personalized Hip Replacement: State of the Art and New Tools Proposals. Proceedings of the 15th International Joint Conference on Biomedical Engineering Systems and Technologies (BIOSTEC 2022).

[12] Kim M.G., Kim J.S., Kim J.J. (2022). 2-D Topology Optimization of the Connection Part of the Electric Kickboard in Case of Front Collision. J. Korean Soc. Precis. Eng..

[13] Akbar M., Aldinger G., Krahmer K., Bruckner T., Aldinger P.R. (2009). Custom Stems for Femoral Deformity in Patients Less than 40 Years of Age: 70 Hips Followed for an Average of 14 Years. Acta Orthop..

[14] Escobar S.B., Bouguecha A., Almohallami A., Niemeier H., Lucas K., Stukenborg-Colsman C., Nolte I., Wefstaedt P., Behrens B.A. (2015). The Customized Artificial Hip Cup: Design and Manufacturing of an Innovative Prosthesis. Lect. Notes Appl. Comput. Mech..

[15] Nam L.T.B., Phuong N.L., Dat N.T., Cuc N.T.K. (2024). Biomechanical Analysis of a Patient-Specific Artificial Hip Joint to Design and Manufacture It by 3D Printing. Proceedings of the 3rd Annual International Conference on Material, Machines and Methods for Sustainable Development (MMMS2022).

[16] Jun Y., Choi K. (2010). Design of Patient-Specific Hip Implants Based on the 3D Geometry of the Human Femur. Adv. Eng. Softw..

[17] Naghavi S.A., Tamaddon M., Garcia-Souto P., Moazen M., Taylor S., Hua J., Liu C. (2023). A Novel Hybrid Design and Modelling of a Customised Graded Ti-6Al-4V Porous Hip Implant to Reduce Stress-Shielding: An Experimental and Numerical Analysis. Front. Bioeng. Biotechnol..

[18] Moscol-Albañil I., Solórzano-Requejo W., Rodriguez C., Ojeda C., Díaz Lantada A. (2024). Innovative AI-Driven Design of Patient-Specific Short Femoral Stems in Primary Hip Arthroplasty. Mater. Des..

[19] Feyen H., Shimmin A.J. (2014). Is the Length of the Femoral Component Important in Primary Total Hip Replacement?. Bone Jt. J..

[20] Samy A.M., El-Tantawy A. (2019). Stem Length in Primary Cementless Total Hip Arthroplasty: Does It Make a Difference in Bone Remodeling?. Eur. J. Orthop. Surg. Traumatol..

[21] Noyama Y., Miura T., Ishimoto T., Itaya T., Niinomi M., Nakano T. (2012). Bone Loss and Reduced Bone Quality of the Human Femur after Total Hip Arthroplasty under Stress-Shielding Effects by Titanium-Based Implant. Mater. Trans..

[22] Reimeringer M., Nuño N. (2016). The Influence of Contact Ratio and Its Location on the Primary Stability of Cementless Total Hip Arthroplasty: A Finite Element Analysis. J. Biomech..

[23] Kang J., Dong E., Li D., Dong S., Zhang C., Wang L. (2020). Anisotropy Characteristics of Microstructures for Bone Substitutes and Porous Implants with Application of Additive Manufacturing in Orthopaedic. Mater. Des..

[24] Sabatini A.L., Goswami T. (2008). Hip Implants VII: Finite Element Analysis and Optimization of Cross-Sections. Mater. Des..

[25] Carballido-Gamio J., Nicolella D.P. (2013). Computational Anatomy in the Study of Bone Structure. Curr. Osteoporos. Rep..

[26] Abdelaal O., Darwish S., El-Hofy H., Saito Y. (2019). Patient-Specific Design Process and Evaluation of a Hip Prosthesis Femoral Stem. Int. J. Artif. Organs.

[27] Krug R., Burghardt A.J., Majumdar S., Link T.M. (2010). High-Resolution Imaging Techniques for the Assessment of Osteoporosis. Radiol. Clin. N. Am..

[28] Huang Y.J., Huang Y.C., Wang S.C., Ku M.C. (2019). Computed Tomography System for Total Knee Replacement Planning and Comparison with Plain Radiography Measured by an Orthopedic Surgeon. J. Imaging Sci. Technol..

[29] Chu C., Chen C., Liu L., Zheng G. (2015). FACTS: Fully Automatic CT Segmentation of a Hip Joint. Ann. Biomed. Eng..

[30] Wiese T., Yao J., Burns J.E., Summers R.M. (2012). Detection of Sclerotic Bone Metastases in the Spine Using Watershed Algorithm and Graph Cut. Proc. SPIE.

[31] Lamecker H., Seebass M., Hege H.-C., Deuflhard P., Lamecker H., Seebass M., Hege H.-C., Deuflhard P. (2004). A 3D Statistical Shape Model of the Pelvic Bone for Segmentation. SPIE.

[32] Ehrhardt J., Handels H., Malina T., Strathmann B., Plötz W., Pöppl S.J. (2001). Atlas-Based Segmentation of Bone Structures to Support the Virtual Planning of Hip Operations. Int. J. Med. Inform..

[33] Abdel-Wahab A.A., Maligno A.R., Silberschmidt V.V. (2012). Micro-Scale Modelling of Bovine Cortical Bone Fracture: Analysis of Crack Propagation and Microstructure Using X-FEM. Comput. Mater. Sci..

[34] Brown T.D., Pedersen D.R., Gray M.L., Brand R.A., Rubin C.T. (1990). Toward an Identification of Mechanical Parameters Initiating Periosteal Remodeling: A Combined Experimental and Analytic Approach. J. Biomech..

[35] Carter D.R. (1984). Mechanical Loading Histories and Cortical Bone Remodeling. Calcif. Tissue Int..

[36] Huiskes R., Rulmerman R., Van Lenthe G.H., Janssen J.D. (2000). Effects of Mechanical Forces on Maintenance and Adaptation of Form in Trabecular Bone. Nature.

[37] Turner C.H. (1998). Three Rules for Bone Adaptation to Mechanical Stimuli. Bone.

[38] Vahdati A., Roubi G., Ghalichi F., Tahani M. (2008). Mechanically Induced Trabecular Bone Remodeling Including Cellular Accommodation Effect: A Computer Simulation. Trans. Can. Soc. Mech. Eng..

[39] Vahdati A., Rouhi G. (2009). A Model for Mechanical Adaptation of Trabecular Bone Incorporating Cellular Accommodation and Effects of Microdamage and Disuse. Mech. Res. Commun..

[40] Rouhi G., Epstein M., Sudak L., Herzog W. (2007). Modeling Bone Resorption Using Mixture Theory with Chemical Reactions. J. Mech. Mater. Struct..

[41] Haase K., Rouhi G. (2013). Prediction of Stress Shielding around an Orthopedic Screw: Using Stress and Strain Energy Density as Mechanical Stimuli. Comput. Biol. Med..

[42] Cowin S.C. (1986). Wolff’s Law of Trabecular Architecture at Remodeling Equilibrium. J. Biomech. Eng..

[43] Raut P., Kawade D.M.M., Waghmare P. (2024). Finite Element Analysis of Femur Bone Exploring Different Loading Conditions and Modelling Fracture Scenarios. Int. J. Res. Publ. Rev..

[44] Cheng X., Yang Y., Zhu J., Li G., Chen W., Wang J., Zhang Q., Zhang Y. (2023). Finite Element Analysis of Basicervical Femoral Neck Fracture Treated with Proximal Femoral Bionic Nail. J. Orthop. Surg. Res..

[45] Chi W.M., Lin C.C., Ho Y.J., Lin H.C., Chen J.H. (2018). Using Nonlinear Finite Element Models to Analyse Stress Distribution during Subluxation and Torque Required for Dislocation of Newly Developed Total Hip Structure after Prosthetic Impingement. Med. Biol. Eng. Comput..

[46] Zhang B., Rao S., Mekkawy K.L., Rahman R., Sarfraz A., Hollifield L., Runge N., Oni J.K. (2023). Risk Factors for Pain after Total Hip Arthroplasty: A Systematic Review. Arthroplasty.

[47] Belwanshi M., Jayaswal P., Aherwar A. (2022). A Study on Finite Element Analysis Methodologies and Approaches Used for Total Hip Arthroplasty. Mater. Today Proc..

[48] Guo L., Ataollah Naghavi S., Wang Z., Nath Varma S., Han Z., Yao Z., Wang L., Wang L., Liu C. (2022). On the Design Evolution of Hip Implants: A Review. Mater. Des..

[49] Jang I.G., Kim I.Y. (2008). Computational Study of Wolff’s Law with Trabecular Architecture in the Human Proximal Femur Using Topology Optimization. J. Biomech..

[50] Khanuja H.S., Vakil J.J., Goddard M.S., Mont M.A. (2011). Cementless Femoral Fixation in Total Hip Arthroplasty. J. Bone Jt. Surg. Am..

[51] Kim J.J., Nam J., Jang I.G. (2018). Computational Study of Estimating 3D Trabecular Bone Microstructure for the Volume of Interest from CT Scan Data. Int. J. Numer. Methods Biomed. Eng..

[52] Beaupré G.S., Orr T.E., Carter D.R. (1990). An Approach for Time-Dependent Bone Modeling and Remodeling--Theoretical Development. J. Orthop. Res..

[53] Tsubota K.I., Adachi T., Tomita Y. (2002). Functional Adaptation of Cancellous Bone in Human Proximal Femur Predicted by Trabecular Surface Remodeling Simulation toward Uniform Stress State. J. Biomech..

[54] Han S., Kim R.S., Harris J.D., Noble P.C. (2019). The Envelope of Active Hip Motion in Different Sporting, Recreational, and Daily-Living Activities: A Systematic Review. Gait Posture.

[55] Noroozi M., Mohammadi H., Efatinasab E., Lashgari A., Eslami M., Khan B. (2022). Golden Search Optimization Algorithm. IEEE Access.

[56] Hildebrand T., Rüegsegger P. (1997). Quantification of Bone Microarchitecture with the Structure Model Index. Comput. Methods Biomech. Biomed. Engin..

[57] Wiik A.V., Aqil A., Al-Obaidi B., Brevadt M., Cobb J.P. (2021). The Impact of Reducing the Femoral Stem Length in Total Hip Arthroplasty during Gait. Arch. Orthop. Trauma Surg..

[58] Davidson D., Pike J., Garbuz D., Duncan C.P., Masri B.A. (2008). Intraoperative Periprosthetic Fractures during Total Hip Arthroplasty: Evaluation and Management. J. Bone Jt. Surg..

[59] Li J., Gong H. (2021). Fatigue Behavior of Cortical Bone: A Review. Acta Mech. Sin..

[60] Reimeringer M., Nuño N., Desmarais-Trépanier C., Lavigne M., Vendittoli P.A. (2013). The Influence of Uncemented Femoral Stem Length and Design on Its Primary Stability: A Finite Element Analysis. Comput. Methods Biomech. Biomed. Eng..

[61] Savio D., Bagno A. (2022). When the Total Hip Replacement Fails: A Review on the Stress-Shielding Effect. Processes.

[62] Turner A.W.L., Gillies R.M., Sekel R., Morris P., Bruce W., Walsh W.R. (2005). Computational Bone Remodelling Simulations and Comparisons with DEXA Results. J. Orthop. Res..

[63] Park C.-W., Lim S.-J., Park Y.-S. (2018). Modular Stems: Advantages and Current Role in Primary Total Hip Arthroplasty. Hip Pelvis.

[64] Mirza S.B., Dunlop D.G., Panesar S.S., Naqvi S.G., Gangoo S., Salih S. (2010). Basic Science Considerations in Primary Total Hip Replacement Arthroplasty. Open Orthop. J..

